# Genomic data assimilation using a higher moment filtering technique for restoration of gene regulatory networks

**DOI:** 10.1186/s12918-015-0154-2

**Published:** 2015-03-13

**Authors:** Takanori Hasegawa, Tomoya Mori, Rui Yamaguchi, Teppei Shimamura, Satoru Miyano, Seiya Imoto, Tatsuya Akutsu

**Affiliations:** Bioinformatics Center, Institute for Chemical Research, Kyoto University, Gokasho, Kyoto, 611-0011 Uji Japan; Human Genome Center, The Institute of Medical Science, The University of Tokyo, 4-6-1 Shirokanedai, Tokyo, 108-8639 Minato-ku Japan; Division of Systems Biology, Nagoya University Graduate School of Medicine, 65 Tsurumai-cho, Nagoya, 466-8550 Showa-ku Japan

**Keywords:** Gene regulatory networks, Time series analysis, Systems biology, Data assimilation, Monte Carlo

## Abstract

**Background:**

As a result of recent advances in biotechnology, many findings related to intracellular systems have been published, *e.g.*, transcription factor (TF) information. Although we can reproduce biological systems by incorporating such findings and describing their dynamics as mathematical equations, simulation results can be inconsistent with data from biological observations if there are inaccurate or unknown parts in the constructed system. For the completion of such systems, relationships among genes have been inferred through several computational approaches, which typically apply several abstractions, *e.g.*, linearization, to handle the heavy computational cost in evaluating biological systems. However, since these approximations can generate false regulations, computational methods that can infer regulatory relationships based on less abstract models incorporating existing knowledge have been strongly required.

**Results:**

We propose a new data assimilation algorithm that utilizes a simple nonlinear regulatory model and a state space representation to infer gene regulatory networks (GRNs) using time-course observation data. For the estimation of the hidden state variables and the parameter values, we developed a novel method termed a higher moment ensemble particle filter (HMEnPF) that can retain first four moments of the conditional distributions through filtering steps. Starting from the original model, *e.g.*, derived from the literature, the proposed algorithm can sequentially evaluate candidate models, which are generated by partially changing the current best model, to find the model that can best predict the data. For the performance evaluation, we generated six synthetic data based on two real biological networks and evaluated effectiveness of the proposed algorithm by improving the networks inferred by previous methods. We then applied time-course observation data of rat skeletal muscle stimulated with corticosteroid. Since a corticosteroid pharmacogenomic pathway, its kinetic/dynamics and TF candidate genes have been partially elucidated, we incorporated these findings and inferred an extended pathway of rat pharmacogenomics.

**Conclusions:**

Through the simulation study, the proposed algorithm outperformed previous methods and successfully improved the regulatory structure inferred by the previous methods. Furthermore, the proposed algorithm could extend a corticosteroid related pathway, which has been partially elucidated, with incorporating several information sources.

**Electronic supplementary material:**

The online version of this article (doi:10.1186/s12918-015-0154-2) contains supplementary material, which is available to authorized users.

## Background

Gene regulatory networks (GRNs) are fundamental for sustaining complex biological systems in cells. Although a comprehensive understanding of intracellular systems is still far from complete, many findings regarding intracellular systems have been published as a result of recent technological advances in biotechnology, *e.g.*, microarray and Chip-Seq. By combining these findings, we can construct biological simulation models in which the dynamics of biomolecules are described by mathematical equations, *e.g.*, the Michaelis-Menten model [1] and S-system [2]. However, simulation results may not match results from biological observations due to inaccurate or missing information about intracellular systems.

In oder to infer unknown parts of biological systems, there exist roughly two major approaches, *i.e.*, simulation model-based and statistical approaches. In constructing biological simulation models, regulatory relationships among biomolecules are collected from the literature. To represent the regulatory systems, mathematical equations, often differential equations [1-4], are given to describe the dynamic behavior of the involved biomolecules. The parameter values of these simulation models have been estimated to be consistent with the data by some computational methodologies. Several methods have been proposed to infer regulatory structures [5,6], to reproduce the dynamic behavior of biological systems recorded in the literature [7-10], and to improve published pathways so that they are consistent with the data [11,12]. However, differential equation-based approaches are computationally intensive when updating parameter values and simulation results simultaneously. Therefore, they cannot be applied to more than several genes when much of the regulatory structure is unknown.

A statistical approach using more abstracted models, *e.g.*, Bayesian networks [13-16] and the state space model [17-21], have been successfully applied to infer the structure of transcriptional regulation using data from biological observations. Whereas purely data-driven methods need to explore a large model space, some studies have further incorporated other information, *e.g.*, literature-recorded pathways and TFs information [22-26]. In contrast, these approximations can generate false regulations; there is a trade-off relationship between accuracy and computational ease. To overcome the problem, methods to improve and deconvolve networks, which are inferred by some computational approaches, utilizing less abstract models to better predict the data have been also proposed recently [27-29]. In following the direction, we should apply models that can maximally emulate the nonlinear dynamics of gene regulatory networks and establish a method for estimating the parameter values that maximize the ability to predict the data.

For this purpose, we proposed a novel data assimilation algorithm utilizing a simple nonlinear model, termed the combinatorial transcription model [5,30], and a state space representation [31,32], to infer GRNs by restoring networks that is inferred by some GRNs inference methods or derived from the literature. Since the nonlinearity results in generating non-Gaussian conditional distributions of the hidden state variables, we applied the unscented Kalman filter (UKF) [33-35] that can efficiently calculate the approximated conditional distributions as Gaussian distributions [36]. However, UKF cannot satisfy the requirements for estimating accurate parameter values of the model; thus, the first four moments of the conditional distributions of the hidden states should be retained. To address this problem, we developed a novel method, termed a higher-moment ensemble particle filter (HMEnPF), which can retain the first two moments and the third and fourth central moments throughout the prediction, filtering, and smoothing steps. Starting from an original network, which is derived from the literature or some GRNs inference methods, the proposed algorithm using HMEnPF improves the network based on the nonlinear state space model. Furthermore, the combinatorial transcription model was extended so that the model can handle additional biomolecules such as drugs.

To show the effectiveness of the proposed algorithm, we first prepared synthetic time-course data and compared the proposed algorithm to GeneNet [37,38] based on an empirical graphical Gaussian model (GGM), G1DBN [39] based on dynamic Bayesian networks using first order conditional dependencies, and the previous algorithm using UKF only [36]. For this comparison, six synthetic data with 30 time-points were generated based on a WNT5A [40] and a yeast-cell-cycle network [41]. As an application example, we used the time-course microarray data after stimulating rat skeletal muscle with corticosteroid, which were downloaded from the GEO database (GSE490). For this experiment, we also utilized corticosteroid pharmacogenomics [42,43], a previously defined regulatory structure for rat skeletal muscle [44], TF information from ITFP (Integrated Transcription Factor Platform) [45] and the original network inferred by G1DBN. Consequently, we proposed candidate pathways for an extension of corticosteroid-related pathways.

## Methods

### State space representation of combinatorial transcription model

Let *x*_*i*_(*t*) be the abundance of the *i*th (*i*=1,…,*p*) gene as a function of time *t*. As a gene regulatory system, we assume that *x*_*i*_(*t*) is controlled by its synthesis and degradation processes, and that the quantity of synthesis is regulated by the other genes as described by

(1) $$ \frac{{dx}_{i}(t)}{dt} = f_{i}\left(\boldsymbol{x}(t), \boldsymbol{\theta}_{f_{i}}\right)\cdot u_{i} - x_{i}(t)\cdot d_{i} + v(t),   $$

where *f*_*i*_ is a function of the regulatory effect on the *i*th gene by other genes, ***x***(*t*)=(*x*_1_(*t*),…,*x*_*p*_(*t*))^′^, $\boldsymbol {\theta }_{f_{i}}$ is a vector of tuning parameters for *f*_*i*_, *u*_*i*_ is a synthesis coefficient, *d*_*i*_ is a degradation coefficient and *v*(*t*) is a random system noise. Here, (·)^′^ stands for transposition. *f*_*i*_ is often considered as a hill function, such as the Michaelis-Menten model [1].

Since the estimation of parameter values maximizing prediction ability is a computationally heavy task when using differential equations, difference equations have been typically utilized to analyze biological systems [4,5,17,18,20,21,46]. The impact of such substitution was discussed previously [3,4]. In this study, we handle a simple nonlinear difference equation based on the combinatorial transcription model [5,30,36] described by

(2) $$\begin{array}{*{20}l} x_{i, t + 1} &= (1+a_{i,i})x_{i, t} + \sum_{j \in \mathcal A_{i}} a_{i, j} \cdot x_{j, t}\\ &~~~~+ \sum_{j \in \mathcal A_{i}} \sum_{k \in \mathcal A_{i}\backslash j} b_{i, (j, k)} \cdot x_{j, t} \cdot x_{k, t} + u_{i} + v_{i, t},  \end{array} $$

where *x*_*i*,*t*_ is the amount of the *i*th gene at time *t*, *a*_*i*,*j*_ is an individual effect by the *j*th gene on the *i*th gene, *b*_*i*,(*j*,*k*)_ is a combinatorial effect of the *j*th and *k*th genes on the *i*th gene and $\mathcal A_{i}$ is an active set of genes regulating the *i*th gene. Since this model is a simple extension of a linear model to express a combinatorial effect by two different genes, *b*_*j*,*j*_ is not considered.

Under the framework of data assimilation, in order to combine the simulation results with the observed experimental data, we apply a state space representation of Eq. (2) given by

(3) $$\begin{array}{*{20}l} \boldsymbol{x}_{t+1} &= A\boldsymbol{x}_{t} + B \text{vec}(\boldsymbol{x}_{t} \boldsymbol{x}_{t}') + \boldsymbol{u} + \boldsymbol{v}_{t},  \end{array} $$

(4) $$\begin{array}{*{20}l} \boldsymbol{y}_{t} &= \boldsymbol{x}_{t} + \boldsymbol{w}_{t},  \end{array} $$

where ***x***_*t*_=(*x*_1,*t*_,…,*x*_*p*,*t*_)^′^, *A*=(***a***_1_,…,***a***_*p*_)^′^∈*R*^*p*×*p*^ is a linear effect matrix, ***a***_*i*_=(*a*_*i*,1_,…,*a*_*i*,*p*_)^′^ (*i*=1,…,*p*), $B=(\boldsymbol {b}_{1}, \ldots, \boldsymbol {b}_{p})'\in R^{p \times p^{2}}$ is a combinatorial effect matrix, ***b***_*i*_=(*b*_*i*,(1,1)_,…,*b*_*i*,(1,*p*)_,*b*_*i*,(2,1)_,…,*b*_*i*,(*p*,*p*)_)^′^ (*i*=1,…,*p*), vec is a transformation function $(R^{p \times p} \rightarrow R^{p^{2}})$, ***u***=(*u*_1_,…,*u*_*p*_)^′^, and ***v***_*t*_∼*N*(0,*Q*) and ***w***_*t*_∼*N*(0,*R*) are system and observational noises with diagonal covariance matrices, respectively. We define an entire set of time points $\mathcal T=\{1, \ldots, T\}$ and the observed time set $\mathcal {T}_{\textit {obs}}$ ($\mathcal {T}_{\textit {obs}}\subset \mathcal {T}$), and consider $\mathcal {T}_{\textit {obs}}=\mathcal {T}$ in the following for simplicity. Note that *A* and *B* should be sparse matrices, and we also consider an active set of elements ${\mathcal B_{i}}(i=1, \ldots, p)$, which are sets of non-zero columns in the *i*th row of *B*.

### Incorporation of biomolecules affecting biological systems

**Figure 1:**
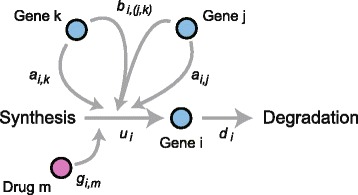
**A combinatorial transcription model.** A cartoon figure of the combinatorial transcription model regarding the *i*th gene. A gene undergoes synthesis and degradation processes, and its synthesis process is regulated through individual effects *a*_*i*,*j*_,*a*_*i*,*k*_ and a combinatorial effect *b*_*i*,(*j*,*k*)_.

Although the regulatory system of Eqs. (3) and (4) can only represent dynamic regulation among genes, other biomolecules, such as drugs, can affect the regulatory system. To address these cases, we further consider a term representing the concentration of other biomolecules as represented by

(5) $$\begin{array}{*{20}l} \boldsymbol{x}_{t} &= A \boldsymbol{x}_{t-1}+ B \text{vec}(\boldsymbol{x}_{t} \boldsymbol{x}_{t}') + G\boldsymbol{d}_{t-1} + \boldsymbol{u} + \boldsymbol{v}_{t},  \end{array} $$

where ***d***_*t*_ is an *M*-dimensional vector containing the concentration of the biomolecules at the *t*th time point, *G*=(***g***_1_,…,***g***_*p*_)^′^ is an *p*×*M* matrix and ***g***_*i*_=(*g*_*i*,1_,…,*g*_*i*,*M*_)^′^ (*i*=1,…,*p*) is an *M*-dimensional vector representing their regulatory effects on the *i*th gene. As with ${\mathcal A}_{i}$ and ${\mathcal B}_{i}$, we consider an active set of elements ${\mathcal G}_{i}$ for the *i*th row of the drug effect *G*. A conceptual view of Eq. (5) is illustrated in Figure 1. In using Eqs. (4) and (5), we try to infer the regulatory structure among genes and estimate the values of ***θ***={*A*, *B*, *G*, ***u***, *Q*, *R*, ***μ***_0_}.

### A higher-moment ensemble particle filter

Let *Y*_*t*_ be {***y***_1_,…,***y***_*t*_}. In estimating the parameter values and calculating the likelihood of Eqs. (4) and (5), conditional probability densities *p*(***x***_*t*_|*Y*_*t*−1_), *p*(***x***_*t*_|*Y*_*t*_) and *p*(***x***_*t*_|*Y*_*T*_) can be non-Gaussian forms. Thus, since these probability densities can be analytically intractable, we applied a type of Monte Carlo approach termed ensemble approximation. In this approach, for example, a probability density *p*(***x***_*t*_) is approximated by

(6) $$\begin{array}{*{20}l} p(\boldsymbol{x}_{t}) = \frac{1}{N}\sum^{N}_{n=1} \delta\left(\boldsymbol{x}_{t} - \boldsymbol{x}_{t}^{(n)}\right), \end{array} $$

where $\boldsymbol {x}_{t}^{(n)}$ is the *n*th sample from *p*(***x***_*t*_), *N* is the number of samples and *δ* is a Dirac delta function. A sample $\boldsymbol {x}_{t}^{(n)}$ and a set of samples $\{\boldsymbol {x}_{t}^{(n)}\}$ are called particle and ensemble, respectively. Previously, many types of ensemble approximation methods have been developed to obtain conditional distributions of the hidden state variables in nonlinear state space models, *e.g.*, the ensemble Kalman filter (EnKF) [47] and the particle filter (PF) [48,49]. Here, our requirements for this study are the followings; (i) the number of particles is not reduced through filtering steps since *p* and the dimension of ***θ*** can be too high for the resampling procedure and (ii) the third and fourth moments of probability densities of the hidden states should be kept to optimize ***θ*** as explained in the next sub-section. To satisfy these requirements, we extended a method termed the ensemble particle filter (EnPF) [50,51], which can retain the first two moments through filtering steps, and developed a novel method termed a higher-moment ensemble particle filter (HM-EnPF) that can additionally retain third and fourth central moments without reducing particles. The procedure of the proposed method is explained below.

#### Prediction step

In this step, we attempt to calculate *p*(***x***_*t*+1_|*Y*_*t*_) after obtaining *p*(***x***_*t*_|*Y*_*t*_) (*t*=0,…,*T*−1). Let $\boldsymbol {x}_{t|t}^{(n)}$ be a sample from a conditional probability density *p*(***x***_*t*_|*Y*_*t*_). Initially, generate particles $\boldsymbol {x}_{0|0}^{(n)} \sim p(\boldsymbol {x}_{0})$ for *n*=1,…,*N*. Then, for *t*=0,…,*T*−1,

1. Generate particles $\boldsymbol {v}_{t}^{(n)}\sim N(0, Q)$ for *n*=1,…,*N*.
2. Calculate $\boldsymbol {x}_{t+1|t}^{(n)}$ by applying $\boldsymbol {x}_{t|t}^{(n)}$ and $\boldsymbol {v}_{t}^{(n)}$ to Eq. (5) for *n*=1,…,*N*.

#### Filtering step

In this step, we attempt to calculate *p*(***x***_*t*+1_|*Y*_*t*+1_) after obtaining *p*(***x***_*t*+1_|*Y*_*t*_) (*t*=0,…,*T*−1). This step consists of the following three sub-steps termed “Particle Filter Step”, “Ensemble Kalman Filter Step” and “Merging Step”.

At the *t*th ($t \in {\mathcal T}_{\textit {obs}}$) time step,

1. “Particle Filter Step” is firstly executed to obtain $\left \{\hat {\boldsymbol {x}}_{t|t}^{(n)}\right \}$ that is according to the theoretically exact conditional probability density *p*(***x***_*t*_|***y***_*t*_) as follows.
  - Resample $\hat {\boldsymbol {x}}_{t|t}^{(n)}$ according to
    (7) $$\begin{array}{*{20}l} p(\boldsymbol{x}_{t}|\boldsymbol{y}_{t}) &= \frac{1}{\sum_{\dot{n}=1}^{N}p\left(\boldsymbol{y}_{t}|\boldsymbol{x}_{t|t-1}^{(\dot{n})}\right)}\\ &~~~~~ \times \sum_{n=1}^{N}p\left(\boldsymbol{y}_{t}|\boldsymbol{x}_{t|t-1}^{(n)}\right)\delta\left(\boldsymbol{x}_{t}-\boldsymbol{x}_{t|t-1}^{(n)}\right). \end{array} $$
  - Calculate the first and second moments $\mu _{t|t} = E\left [\left \{\hat {\boldsymbol {x}}_{t|t}^{(n)}\right \}\right ]$ and $V_{t|t}=Var\left [\left \{\hat {\boldsymbol {x}}_{t|t}^{(n)}\right \}\right ]$, respectively.
  - Standardize $\hat {\boldsymbol {x}}_{t|t}^{(n)}$ as
    (8) $$\begin{array}{*{20}l} \hat{\boldsymbol{z}}_{t|t}^{(n)} = V_{t|t}^{-\frac{1}{2}} \cdot \left(\hat{\boldsymbol{x}}_{t|t}^{(n)} - \boldsymbol{\mu}_{t|t}\right). \end{array} $$
  - Calculate the third and fourth central moments $\hat {\boldsymbol {m}}_{t|t}^{(3)}=E\left [\left \{\hat {\boldsymbol {z}}_{t|t}^{(n)}\right \}^{3}\right ]$ and $\hat {\boldsymbol {m}}_{t|t}^{(4)} =E\left [\left \{\hat {\boldsymbol {z}}_{t|t}^{(n)}\right \}^{4}\right ]$, respectively.
2. “Ensemble Kalman Filter Step” is secondly executed to obtain $\left \{\tilde {\boldsymbol {x}}_{t|t}^{(n)}\right \}$ that is calculated under the Gaussian assumption with regard to *p*(***x***_*t*_|***y***_*t*_) as follows.
  - Generate particles $\boldsymbol {w}_{t}^{(n)}\sim N(0, R)$ for *n*=1,…,*N*.
  - Calculate Kalman gain
    (9) $$\begin{array}{*{20}l} K_{t} = V_{t|t-1}\left(V_{t|t-1} + R_{t}\right)^{-1}, \end{array} $$
    where $V_{t|t-1} = Var\left [\left \{\boldsymbol {x}_{t|t-1}^{(n)}\right \}\right ]$ and $R_{t} = Var\left [\left \{\boldsymbol {w}_{t}^{(n)}\right \}\right ]$.
  - Calculate $\tilde {\boldsymbol {x}}_{t|t}^{(n)}$ as
    (10) $$\begin{array}{*{20}l} \tilde{\boldsymbol{x}}_{t|t}^{(n)} = \boldsymbol{x}_{t|t-1}^{(n)} + K_{t} \left(\boldsymbol{y}_{t} - \boldsymbol{x}_{t|t-1}^{(n)} + \boldsymbol{w}_{t}^{(n)}\right). \end{array} $$
  - Calculate the first and second moments $\tilde {\mu }_{t|t} = E\left [\left \{\tilde {\boldsymbol {x}}_{t|t}^{(n)}\right \}\right ]$ and $\tilde {V}_{t|t}=Var\left [\left \{\tilde {\boldsymbol {x}}_{t|t}^{(n)}\right \}\right ]$, respectively.
  - Standardize $\tilde {\boldsymbol {x}}_{t|t}^{(n)}$ as
    (11) $$\begin{array}{*{20}l} \tilde{\boldsymbol{z}}_{t|t}^{(n)} = \tilde{V}_{t|t}^{-\frac{1}{2}} \cdot \left(\tilde{\boldsymbol{x}}_{t|t}^{(n)}- \tilde{\boldsymbol{\mu}}_{t|t}\right). \end{array} $$
  - Calculate the third and fourth central moments $\tilde {\boldsymbol {m}}_{t|t}^{(3)}=E\left [\left \{\tilde {\boldsymbol {z}}_{t|t}^{(n)}\right \}^{3}\right ]$ and $\tilde {\boldsymbol {m}}_{t|t}^{(4)} =E\left [\left \{\tilde {\boldsymbol {z}}_{t|t}^{(n)}\right \}^{4}\right ]$, respectively.
3. “Merging Step” is finally executed to obtain $\left \{\boldsymbol {x}_{t|t}^{(n)}\right \}$ of which the first, second, third central and fourth central moments match to those of $\left \{\hat {\boldsymbol {x}}_{t|t}^{(n)}\right \}$. Here, we consider a standardization function *S*(***γ***,***α***,***β***) that transforms a normal random vector ***γ*** into a normalized random vector ***x*** whose the third and fourth central moments are ***α*** and ***β***, respectively. From a previous study [52], we have *S*(***γ***,***α***,***β***) and *S*_*inv*_(***x***,***α***,***β***) that transforms ***x*** to ***γ*** as explained in the Additional file 1. Then, we obtained $\boldsymbol {x}_{t|t}^{(n)}$ as
  (12) $$\begin{array}{*{20}l} \boldsymbol{x}_{t|t}^{(n)} &= \hat{V}_{t|t}^{\frac{1}{2}}S\left(\boldsymbol{z}_{t}^{(n)}, \hat{\boldsymbol{m}}_{t|t}^{(3)}, \hat{\boldsymbol{m}}_{t|t}^{(4)}\right) + \hat{\boldsymbol{\mu}}_{t|t}, \end{array} $$
  (13) $$\begin{array}{*{20}l} \boldsymbol{z}_{t}^{(n)} &= S_{inv}\left(\tilde{\boldsymbol{z}}_{t|t}^{(n)}, \tilde{\boldsymbol{m}}_{t|t}^{(3)}, \tilde{\boldsymbol{m}}_{t|t}^{(4)}\right). \end{array} $$

#### Smoothing step

The smoothing step used for calculating ***x***_*t*|*s*_ (*s*>*t*) was essentially the same as the filtering step. The details of the smoothing step can be found in the Additional file 2.

### Parameter estimation using EM-algorithm

Let *X*_*T*_={***x***_0_, …, ***x***_*T*_} be the set of state variables. The log-likelihood of observation data is given by

(14) $$\begin{array}{*{20}l}  \log L=\log\!\int\! p(\boldsymbol x_{0}) \!\prod_{t \in \mathcal{T}} p(\boldsymbol x_{t}|\boldsymbol x_{t-1}) \!\prod_{t \in \mathcal{T}_{obs}}\!p(\boldsymbol y_{t}|\boldsymbol x_{t})d\boldsymbol{x}_{1}\ldots d\boldsymbol{x}_{T},  \end{array} $$

where *p*(***x***_0_) is a probability density of *N*-dimensional Gaussian distributions *N*(***μ***_0_,*Σ*_0_), *p*(***x***_*t*_|***x***_*t*−1_) and *p*(***y***_*t*_|***x***_*t*_) can be probability densities of *N*-dimensional non-Gaussian distributions obtained by ensemble approximation.

In this study, we estimate the values of ***θ*** by maximizing Eq. (14) using the EM-algorithm. Thus, the conditional expectation of the joint log-likelihood of the complete data (*X*_*T*_,*Y*_*T*_) at the *l*th iteration

(15) $$\begin{array}{*{20}l} q(\boldsymbol \theta | \boldsymbol \theta_{l}) = E[\log p(Y_{T}, X_{T} | \boldsymbol \theta) | Y_{T},\boldsymbol \theta_{l}], \end{array} $$

is iteratively maximized with respect to ***θ*** until the convergence is attained. More details are included in the Additional file 3.

### Network restoration algorithm

**Figure 2:**
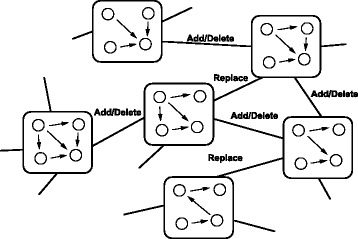
**The schematic view of the proposed algorithm.** The proposed algorithm performs three ways to explore model space, thus, adding, deleting and replacing a regulation from the current best model. Starting from the original model, the proposed algorithm tries to find the best model with respect to the BIC score.

We consider an algorithm to explore the best model by sequentially evaluating candidate models generated from the current best model ${\mathcal M}_{\textit {current}}$ by partially modifying the regulation. Briefly, given the original model ${\mathcal M}_{\textit {original}}$, we attempt to sequentially create and evaluate candidates that are generated by adding, deleting and replacing regulatory components of ${\mathcal M}_{\textit {current}}$ until the best model is no longer updated. A conceptual view is illustrated in Figure 2.

**Figure 3:**
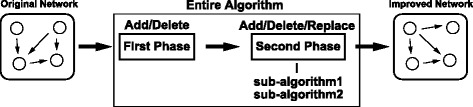
**The analysis flow of the proposed algorithm.** The proposed algorithm (Algorithm 1) consists of two phases (Algorithm 2 and 3) and the second phase consists of two sub-algorithms (Algorithm 4 and 5). Starting from the original model, the proposed algorithm tries to explore the best model.











Due to the heavy computational cost to evaluate the model by HMEnPF, we proposed a novel algorithm for reconstructing GRNs with combining UKF and HMEnPF as described in Algorithms 1, 2, 3, 4 and 5 and illustrated in Figure 3. Compared to EnKF (the computational task of EnKF is included in HMEnPF), the computational cost for UKF in prediction, filtering, and smoothing steps are roughly $\frac {2p+1}{N}$, $\frac {1}{N}$ and $\frac {2}{N\cdot \mathcal {T}_{\textit {obs}}}$, respectively. The theoretical details of UKF for the combinatorial transcription model were discussed previously [36]. Briefly, the proposed algorithm first calculate *e*_*a*_, *e*_*b*_ and *e*_*g*_ explained in the Additional file 4 for all candidate models, next evaluate the top *r*_1_ candidates for each row by UKF and then evaluate the *r*_2_ top candidates by HMEnPF. Note that, when the systems include *G*, regulations by the drugs are inferred in the same way as *A* in Algorithms 1, 2, 3, 4 and 5. In Results and discussion section, we set {*r*_1_,*r*_2_,*add*_*max*_,*del*_*max*_}={5,5,+*∞*,+*∞*}.

## Results and discussion

### Comparison using synthetic data

**Figure 4:**
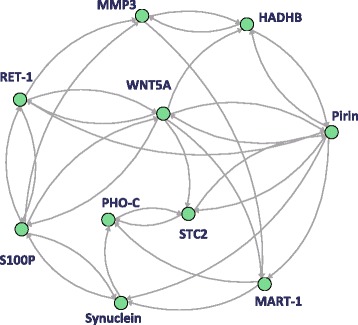
**A WNT5A network.** A real biological network, termed WNT5A network [40], used for the comparison analysis. A node and an arrow represent a gene and regulatory effect, respectively.

**Figure 5:**
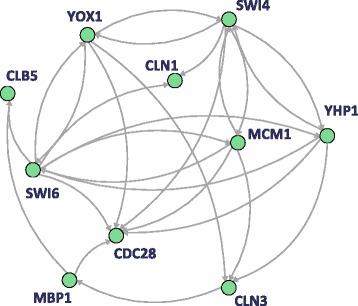
**A yeast cell-cycle network.** A real biological network of yeast cell-cycle from the KEGG database [41] used for the comparison analysis. A node and an arrow represent a gene and regulatory effect, respectively.

To show the effectiveness of the proposed algorithm, we prepared synthetic time-course gene expression data based on the synthetic networks, WNT5A [40] and a yeast cell-cycle [41], as illustrated in Figures 4 and 5, respectively. For each network and three different system noises, we generated five time-courses (${\mathcal T}=\{1,2,\ldots,30\}$) by using Eqs. (3) and (4); thus, six sets of five time-courses were prepared. The values of the parameters *A*, *B* and ***u*** in Eq. (3) were determined between -1 and 1, the system noise ***v***_*t*_ and observational noise ***w***_*t*_ were generated according to Gaussian distributions with a mean 0 and three variances 0.01, 0.05 and 0.1, and that with a mean 0 and a variance 0.3, respectively. For the original networks to be improved by the proposed algorithm, we utilized GeneNet [37,38] based on an empirical graphical Gaussian model (GGM) and G1DBN [39] based on dynamic Bayesian networks using first order conditional dependencies. After the restoration, the original and improved networks were evaluated by true positive (TP), false positive (FP), true negative (TN), false negative (FN), precision rate $\left (PR=\frac {\text {TP}}{\text {TP}+\text {FP}}\right)$, recall rate $\left (RR={\frac {\text {TP}}{\text {{TP}+\text {FN}}}}\right)$ and F-measure $\left (=\frac {\text {2PR}\cdot \text {RR}}{\text {PR}+\text {RR}}\right)$. Note that, since GeneNet infers undirected regulations among genes, we compared its results to the undirected true networks. In addition, since a directed network is required for the original network, we transformed the undirected network inferred by GeneNet as follows; (i) a true directed regulation was set when an inferred undirected regulation was correct, and (ii) a false directed regulation of which direction was randomly selected was set when an inferred undirected regulation was incorrect. Here, to clarify the significance of HMEnPF, we also showed the results of the previous algorithm using UKF only [36]. These results are summarized in Tables 1, 2, 3, 4, 5, 6, 7, 8, 9, 10, 11 and 12.

**Table 1:** **The comparison results using the original model given by G1DBN and the five time-courses generated from WNTA5A network with Gaussian system noise of mean 0 and variance 0.01**

	**PR**	**RR**	**F-measure**	**TP**	**FP**	**TN**	**FN**
HMEnPF	0.689	0.573	0.625	17.2	7.8	52.2	12.8
UKF	0.632	0.533	0.577	16.0	9.4	50.6	14.0
G1DBN	0.487	0.453	0.466	13.6	15.0	45.0	16.4

The comparison results of the proposed algorithm, the previous algorithm using UKF only [36], and G1DBN for the five time-courses generated from WNTA5A network with Gaussian system noise of mean 0 and variance 0.01. The results of ‘PR’, ‘RR’, ‘F-measure’, ‘TP’, ‘FP’, ‘TN’ and ‘FN’ for the five time-courses were averaged. The networks inferred by G1DBN were used as the original networks for the former two algorithms.

**Table 2:** **The comparison results using the original model given by GeneNet and the five time-courses generated from WNTA5A network with Gaussian system noise of mean 0 and variance 0.01**

	**PR**	**RR**	**F-measure**	**TP**	**FP**	**TN**	**FN**
HMEnPF	0.595	0.520	0.553	15.6	10.4	49.6	14.4
UKF	0.573	0.493	0.529	14.8	10.6	49.4	15.2
GeneNet	0.493	0.495	0.493	10.4	10.8	13.2	10.6

The comparison results of the proposed algorithm, the previous algorithm using UKF only [36], and GeneNet for the five time-courses generated from WNTA5A network with Gaussian system noise of mean 0 and variance 0.01. The results of ‘PR’, ‘RR’, ‘F-measure’, ‘TP’, ‘FP’, ‘TN’ and ‘FN’ for the five time-courses were averaged. The networks inferred by GeneNet were used as the original networks for the former two algorithms.

**Table 3:** **The comparison results using the original model given by G1DBN and the five time-courses generated from a yeast-cell cycle network with Gaussian system noise of mean 0 and variance 0.01**

	**PR**	**RR**	**F-measure**	**TP**	**FP**	**TN**	**FN**
HMEnPF	0.759	0.700	0.727	18.2	5.8	58.2	7.8
UKF	0.707	0.692	0.698	18.0	7.6	56.4	8.0
G1DBN	0.574	0.562	0.555	14.6	12.8	51.2	11.4

The comparison results of the proposed algorithm, the previous algorithm using UKF only [36], and G1DBN for the five time-courses generated from a yeast cell-cycle network with Gaussian system noise of mean 0 and variance 0.01. The results of ‘PR’, ‘RR’, ‘F-measure’, ‘TP’, ‘FP’, ‘TN’ and ‘FN’ for the five time-courses were averaged. The networks inferred by G1DBN were used as the original networks for the former two algorithms.

**Table 4:** **The comparison results using the original model given by GeneNet and the five time-courses generated from a yeast cell-cycle network with Gaussian system noise of mean 0 and variance 0.01**

	**PR**	**RR**	**F-measure**	**TP**	**FP**	**TN**	**FN**
HMEnPF	0.827	0.738	0.778	19.2	4.2	59.8	6.8
UKF	0.703	0.708	0.704	18.4	8.0	56.0	7.6
GeneNet	0.413	0.520	0.460	10.4	14.8	10.2	9.6

The comparison results of the proposed algorithm, the previous algorithm using UKF only [36], and GeneNet for the five time-courses generated from a yeast cell-cycle network with Gaussian system noise of mean 0 and variance 0.01. The results of ‘PR’, ‘RR’, ‘F-measure’, ‘TP’, ‘FP’, ‘TN’ and ‘FN’ for the five time-courses were averaged. The networks inferred by GeneNet were used as the original networks for the former two algorithms.

**Table 5:** **The comparison results using the original model given by G1DBN and the five time-courses generated from WNTA5A network with Gaussian system noise of mean 0 and variance 0.05**

	**PR**	**RR**	**F-measure**	**TP**	**FP**	**TN**	**FN**
HMEnPF	0.646	0.580	0.609	17.4	9.4	50.6	12.6
UKF	0.609	0.573	0.589	17.2	10.8	49.2	12.8
G1DBN	0.490	0.460	0.468	13.8	14.6	45.4	16.2

The comparison results of the proposed algorithm, the previous algorithm using UKF only [36], and G1DBN for the five time-courses generated from WNTA5A network with Gaussian system noise of mean 0 and variance 0.05. The results of ‘PR’, ‘RR’, ‘F-measure’, ‘TP’, ‘FP’, ‘TN’ and ‘FN’ for the five time-courses were averaged. The networks inferred by G1DBN were used as the original networks for the former two algorithms.

**Table 6:** **The comparison results using the original model given by GeneNet and the five time-courses generated from WNTA5A network with Gaussian system noise of mean 0 and variance 0.05**

	**PR**	**RR**	**F-measure**	**TP**	**FP**	**TN**	**FN**
HMEnPF	0.705	0.633	0.665	19.0	8.0	52.0	11.0
UKF	0.649	0.567	0.604	17.0	9.2	50.8	13.0
GeneNet	0.453	0.543	0.492	11.4	14.0	10.0	9.6

The comparison results of the proposed algorithm, the previous algorithm using UKF only [36], and GeneNet for the five time-courses generated from WNTA5A network with Gaussian system noise of mean 0 and variance 0.05. The results of ‘PR’, ‘RR’, ‘F-measure’, ‘TP’, ‘FP’, ‘TN’ and ‘FN’ for the five time-courses were averaged. The networks inferred by GeneNet were used as the original networks for the former two algorithms.

**Table 7:** **The comparison results using the original model given by G1DBN and the five time-courses generated from a yeast-cell cycle network with Gaussian system noise of mean 0 and variance 0.05**

	**PR**	**RR**	**F-measure**	**TP**	**FP**	**TN**	**FN**
HMEnPF	0.661	0.600	0.628	15.6	8.0	56.0	10.4
UKF	0.573	0.538	0.553	14.0	10.4	53.6	12.0
G1DBN	0.482	0.515	0.495	13.4	15.0	49.0	12.6

The comparison results of the proposed algorithm, the previous algorithm using UKF only [36], and G1DBN for the five time-courses generated from a yeast cell-cycle network with Gaussian system noise of mean 0 and variance 0.05. The results of ‘PR’, ‘RR’, ‘F-measure’, ‘TP’, ‘FP’, ‘TN’ and ‘FN’ for the five time-courses were averaged. The networks inferred by G1DBN were used as the original networks for the former two algorithms.

**Table 8:** **The comparison results using the original model given by GeneNet and the five time-courses generated from a yeast cell-cycle network with Gaussian system noise of mean 0 and variance 0.05**

	**PR**	**RR**	**F-measure**	**TP**	**FP**	**TN**	**FN**
HMEnPF	0.604	0.577	0.589	15.0	9.8	54.2	11.0
UKF	0.578	0.562	0.568	14.6	11.0	53.0	11.4
GeneNet	0.387	0.360	0.366	7.2	11.2	13.8	12.8

The comparison results of the proposed algorithm, the previous algorithm using UKF only [36], and GeneNet for the five time-courses generated from a yeast cell-cycle network with Gaussian system noise of mean 0 and variance 0.05. The results of ‘PR’, ‘RR’, ‘F-measure’, ‘TP’, ‘FP’, ‘TN’ and ‘FN’ for the five time-courses were averaged. The networks inferred by GeneNet were used as the original networks for the former two algorithms.

**Table 9:** **The comparison results using the original model given by G1DBN and the five time-courses generated from WNTA5A network with Gaussian system noise of mean 0 and variance 0.1**

	**PR**	**RR**	**F-measure**	**TP**	**FP**	**TN**	**FN**
HMEnPF	0.689	0.633	0.659	19.0	8.8	51.2	11.0
UKF	0.637	0.600	0.616	18.0	10.6	49.4	12.0
G1DBN	0.590	0.513	0.548	15.4	11.0	49.0	14.6

The comparison results of the proposed algorithm, the previous algorithm using UKF only [36], and G1DBN for the five time-courses generated from WNTA5A network with Gaussian system noise of mean 0 and variance 0.1. The results of ‘PR’, ‘RR’, ‘F-measure’, ‘TP’, ‘FP’, ‘TN’ and ‘FN’ for the five time-courses were averaged. The networks inferred by G1DBN were used as the original networks for the former two algorithms.

**Table 10:** **The comparison results using the original model given by GeneNet and the five time-courses generated from WNTA5A network with Gaussian system noise of mean 0 and variance 0.1**

	**PR**	**RR**	**F-measure**	**TP**	**FP**	**TN**	**FN**
HMEnPF	0.671	0.607	0.635	18.2	9.0	51.0	11.8
UKF	0.644	0.593	0.615	17.8	10.2	49.8	12.2
GeneNet	0.503	0.590	0.542	12.4	12.2	11.8	8.6

The comparison results of the proposed algorithm, the previous algorithm using UKF only [36], and GeneNet for the five time-courses generated from WNTA5A network with Gaussian system noise of mean 0 and variance 0.1. The results of ‘PR’, ‘RR’, ‘F-measure’, ‘TP’, ‘FP’, ‘TN’ and ‘FN’ for the five time-courses were averaged. The networks inferred by GeneNet were used as the original networks for the former two algorithms.

**Table 11:** **The comparison results using the original model given by G1DBN and the five time-courses generated from a yeast-cell cycle network with Gaussian system noise of mean 0 and variance 0.1**

	**PR**	**RR**	**F-measure**	**TP**	**FP**	**TN**	**FN**
HMEnPF	0.721	0.731	0.725	19.0	7.4	56.6	7.0
UKF	0.714	0.715	0.713	18.6	7.6	56.4	7.4
G1DBN	0.611	0.585	0.591	15.2	10.2	53.8	10.8

The comparison results of the proposed algorithm, the previous algorithm using UKF only [36], and G1DBN for the five time-courses generated from a yeast cell-cycle network with Gaussian system noise of mean 0 and variance 0.1. The results of ‘PR’, ‘RR’, ‘F-measure’, ‘TP’, ‘FP’, ‘TN’ and ‘FN’ for the five time-courses were averaged. The networks inferred by G1DBN were used as the original networks for the former two algorithms.

**Table 12:** **The comparison results using the original model given by GeneNet and the five time-courses generated from a yeast cell-cycle network with Gaussian system noise of mean 0 and variance 0.1**

	**PR**	**RR**	**F-measure**	**TP**	**FP**	**TN**	**FN**
HMEnPF	0.724	0.746	0.735	19.4	7.4	56.6	6.6
UKF	0.690	0.731	0.709	19.0	8.6	55.4	7.0
GeneNet	0.407	0.460	0.427	9.2	13.6	11.4	10.8

The comparison results of the proposed algorithm, the previous algorithm using UKF only [36], and GeneNet for the five time-courses generated from a yeast cell-cycle network with Gaussian system noise of mean 0 and variance 0.1. The results of ‘PR’, ‘RR’, ‘F-measure’, ‘TP’, ‘FP’, ‘TN’ and ‘FN’ for the five time-courses were averaged. The networks inferred by GeneNet were used as the original networks for the former two algorithms.

The results indicate that the proposed algorithm using HMEnPF and only UKF could outperform G1DBN and GeneNet, and the proposed algorithm showed better performance than that of using UKF only. This concludes that retaining higher moment information can improve the accuracy of approximation and estimate correct parameter values. Additionally, we recognized that the performance of the proposed algorithm strongly depends on the accuracy of the original network. Thus, to obtain better results, we should carefully construct original networks or select inference methods for creating the original network. Note that the Jar file of the proposed algorithm is available at: http://sunflower.kuicr.kyoto-u.ac.jp/~t-hasegw/, and the synthetic data, the parameter values and the original networks are in the Additional file 5.

### Inference using real data

As an application example, we analyzed microarray time-course gene expression data from rat skeletal muscle [42,43]. The microarray data were downloaded from the GEO database (GSE490). The time-course gene expression data was measured at 0, 0.25, 0.5, 0.75, 1, 2, 4, 5, 5.5, 7, 8, 12, 18, 30, 48, and 72 [h] (16 time points) after stimulation of corticosteroid, but we removed data at 48 and 72 [h] (steady state profiles) for computational efficiency. The data at time 0 represent controls (untreated). There were two, three, or four replicated observations for each time point.

Because corticosteroid pharmacokinetics/dynamics in skeletal muscle have been modeled based on differential equations [43], the time-dependent concentration of corticosteroid in nucleus in rat skeletal muscle can be obtained for ***d***_*t*_ as explained in the Additional file 6. Furthermore, corticosteroid catabolic/anabolic processes in rat skeletal muscle have been partially established [44]; thus, we handled gene (i) TFs, *Trim63*, *Akt1*, *Akt2*, *Mstn*, *Mtor*, *Irs1*, and (ii) non-TFs, *Akt3*, *Anxa3*, *Bcat2*, *Bnip3*, *Foxo1*, *Igf1*, *Igf1r*, *Pik3c3*, *Pik3cb*, *Pik3cd*, *Pik3c2g*, *Rheb*, *Slc2a4* with their regulatory relationships. Additionally, we handled genes (iii) TFs, *Cebpb*, *Cebpd*, *Gpam*, *Srebf1* and (iv) non-TFs, *Rxrg*, *Scarb1*, *Scd*, *Gpd2*, *Mapk6*, *Ace*, *Ptpn1*, *Ptprf*, *Edn1*, *Agtr1a*, *Ppard*, *Hmgcs2*, *Serpine1*, *Il6r*, *Mapk14*, *Ucp3* and *Pdk4* that are suggested as corticosteroid related genes [42]. Note that the microarray (GSE490) does not include three genes in the original pathway [44], *Redd1*, *Bcaa* and *Klf15*. In summary, we handled the concentration of corticosteroid in nucleus, these 40 genes (shown in Table 13) and an original network that was inferred by G1DBN with regulatory relationships among (i) and (ii). Note that TFs information was derived from ITFP (Integrated Transcription Factor Platform) [45].

**Table 13:** **Sets of pharmacogenomic genes handled in the real data experiment**

	**Gene Set**	**Literature**	**TF**
		**[** 43 **]/[** 42 **]**	**candidate**
(i)	*Trim63*, *Akt1*, *Akt2*, *Mstn*, *Irs1*	∘/-	∘
(ii)	*Akt3*, *Anxa3*, *Bcat2*, *Bnip3*,	∘/-	-
	*Foxo1*, *Igf1*, *Igf1r*, *Mtor*		
	*Pik3c3*, *Pik3cb*, *Pik3cd*,		
	*Pik3c2g*, *Rheb*, *Slc2a4*		
(iii)	*Cebpb*, *Cebpd*, *Gpam*, *Srebf1*	-/ ∘	∘
	*Rxrg*, *Scarb1*, *Scd*, *Gpd2*,		
	*Mapk6*, *Ace*, *Ptpn1*		
(iv)	*Ptprf*, *Edn1*, *Agtr1a*, *Ppard*,	-/ ∘	-
	*Hmgcs2*, *Serpine1*		
	*Il6r*, *Mapk14*, *Ucp3*, *Pdk4*		

**Figure 6:**
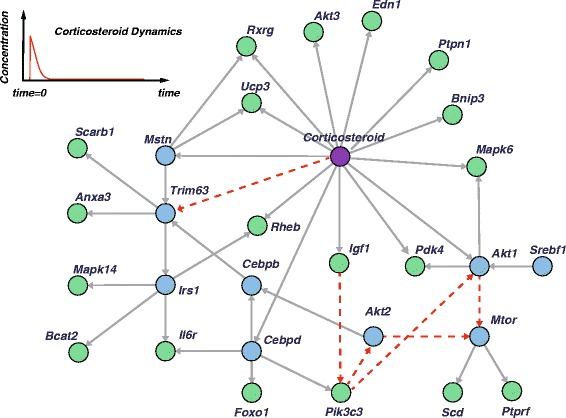
**An inferred network of rat pharmacogenomics by the proposed algorithm.** An inferred network of corticosteroid pharmacogenomics in rat skeletal muscle by the proposed algorithm. Since a part of the pharmacogenomic system has been investigated previously, we inferred the relationships incorporating known pathways (red dotted arrows) and related genes [43 **,** 44], where a purple circle, blues circles and green circles represent corticosterid, TF candidates and non-TF candidates, respectively.

Consequently, we obtained the improved network as illustrated in Figure 6. A purple circle, blues circles, and green circles represent corticosterid, TF candidates and non-TF candidates, respectively. In the center of this figure, there exist corticosteroid regulations to several TF and nonTF genes and regulatory effects transmit to down stream genes of TF candidates genes. In addition, there exist some interesting findings. At first, genes included in ‘response to insulin stimulus (GO:0032868)’ and ‘insulin receptor binding (GO:0005158)’, ‘Igf1’, ‘Akt1’, ‘Akt2’, ‘Srebf1’, ‘Ptprf’, ‘Mtor’ and ‘Ptpn1’, construct a regulatory component in the bottom right of this figure. Including ‘Cebpd’ and ‘Cebpb’, which are assumed to be candidate genes for insulin-related transcription factors and selected as hub genes, functional relationships between corticosteroid and insulin-related functions were reported [53]. On the other hand, ‘Irs1’, ‘Bcat2’, ‘Edn1’, ‘Ucp3’, ‘Pdk4’, ‘Mstn’, ‘Foxo1’ and ‘Rxrg’ that are involved in ‘positive regulation of metabolic process (GO:0009893)’ and ‘fatty acid metabolic process (GO:0006631)’ build the other regulatory process. Since some combinatorial regulations were inferred, it is conceivable that higher moment approximation can affect the estimation results beyond linear models.

## Conclusions

In this paper, we developed a novel approach to restore original GRNs to be consistent with time-course mRNA expression data based on the combinatorial transcription model. Since we applied a state space representation with the nonlinear system equation in the context of data assimilation, the conditional distributions of the hidden variables can be non-Gaussian distributions. In contrast to the previous approaches using particle filter, Gaussian approximation and regression-based solutions, our proposed approach, HMEnPF, can retain the first, second, third central and fourth central moments through filtering steps to estimate near optimal parameter values by the EM-algorithm.

According to the comparison results using six synthetic data based on the real biological pathways, the proposed algorithm successfully explored better models than the previous methods, G1DBN and GeneNet, considering linear relevance. Moreover, the proposed algorithm using HMEnPF outperformed that of using UKF. This concludes that HMEnPF retaining parts of higher moment information can improve the accuracy of the estimation of the parameter values beyond unscented approximation (that cannot retain any moment through filtering steps based on Gaussian approximation). Through the experimental results, we also observed that the performance of the restoration algorithm strongly depends on the original network, which was prepared by literature information or some GRNs inference methods. Thus, one of significant points is to select methods to infer the original network. On the other hand, the proposed method has some limitations. For example, we require time-course data in which the number of time points should be more than 10 or so. Moreover, due to its heavy computational costs, the calculation for more than 20−30 genes without TF information can be infeasible.

As an application example, we prepared corticosteroid pharmacogenomic pathways in rat skeletal muscle that have been investigated and established a part of regulatory relationships and related genes. Additionally, the intracellular concentration of corticosteroid that directly/indirectly affects gene expression can be obtained by the previously developed differential equations and TF information for rat genes can also be utilized. In summary, we inferred the regulatory relationships among 40 genes and corticosteroid with fixing the established pathways and restricting that only TF candidates can regulate other genes. G1DBN was employed to construct the original model for the proposed algorithm. Consequently, several combinatorial regulations and regulations by corticosteroid were inferred by extending the original network. Since previous linear models may not be able to infer these regulations, the proposed algorithm can be valuable to restore inferred and literature-based networks to be consistent with the data.

## Additional files

Additional file 1
**The standardization function for HMEnPF.** The standardization function and its inverse function for HMEnPF are described in the file.

Additional file 2
**The procedure of the smoothing step in HMEnPF.** The procedure of the smoothing step in HMEnPF is described in the file.

Additional file 3
**The detailed solution of the EM-algorithm for the estimation of the parameter values.** The detailed solution of the E- and M-steps in the EM-algorithm for HMEnPF are described in the file.

Additional file 4
**The functions measuring the effectiveness of regulatory edges.** The functions measuring the effectiveness of regulatory edges *e*_*a*_, *e*_*b*_ and *e*_*g*_ are described in the file.

Additional file 5
**The synthetic data from WNT5A and a yeast cell-cycle networks.** The six synthetic data from WNT5A and a yeast cell-cycle networks, their parameter values and the original networks are included in the file.

Additional file 6
**The corticosteroid pharmacokinetics/dynamics in rat skeletal muscle.** The differential equations of rat pharmacokinetics/dynamics are described in the file.
