# Supplementary material for: Genomic data assimilation using a higher moment filtering technique for restoration of gene regulatory networks
Source: BMC Syst Biol. 2015 Mar 13;9:14. doi: 10.1186/s12918-015-0154-2 (PMC4371723; doi:10.1186/s12918-015-0154-2)
Supplement: Additional file 1 — The standardization function for HMEnPF. The standardization function and its inverse function for HMEnPF are described in the file. [file 12918_2015_154_MOESM1_ESM.pdf]

Additional File 1

The Standardizations Function Used for HMEnPF

We consider a standardization function $S(\boldsymbol{\gamma} = (\gamma_1, \ldots, \gamma_p)', \boldsymbol{\alpha} = (\alpha_1, \ldots, \alpha_p)', \boldsymbol{\beta} = (\beta_1, \ldots, \beta_p)')$ that transforms a p-dimensional normal random vector $\boldsymbol{\gamma}$ into a p-dimensional normalized random vector $\boldsymbol{x} = (x_1, \ldots, x_p)'$ where $\boldsymbol{\alpha} = (E[(x_1 - E[x_1])^3], \ldots, E[(x_p - E[x_p])^3])'$ and $\boldsymbol{\beta} = (E[(x_1 - E[x_1])^4], \ldots, E[(x_p - E[x_p])^4])'$, respectively. From a previous study [1], we have $S(\boldsymbol{\gamma}, \boldsymbol{\alpha}, \boldsymbol{\beta})$ and $S_{inv}(\boldsymbol{x}, \boldsymbol{\alpha}, \boldsymbol{\beta})$ that transforms $\boldsymbol{x}$ to $\boldsymbol{\gamma}$. These functions are described by

$$S(\boldsymbol{\gamma}, \boldsymbol{\alpha}, \boldsymbol{\beta}) = (s(\gamma_1, \alpha_1, \beta_1), \ldots, s(\gamma_p, \alpha_p, \beta_p))', \tag{S3-1}$$

$$s(\gamma, \alpha, \beta) = -l_1 + k_1\gamma + l_1\gamma^2 + k_2\gamma^3, \tag{S3-2}$$

$$l_1 = \frac{\alpha}{6(1 + 6l_2)}, \tag{S3-3}$$

$$l_2 = \frac{1}{36}(\sqrt{6\beta - 8\alpha^2 - 14} - 2), \tag{S3-4}$$

$$k_1 = \frac{1 - 3l_2}{1 + l_1^2 - l_2^2}, \tag{S3-5}$$

$$k_2 = \frac{l_2}{(1 + l_2^2 + 12l_2^2)}, \tag{S3-6}$$

and

$$S_{inv}(\boldsymbol{x}, \boldsymbol{\alpha}, \boldsymbol{\beta}) = (s_{inv}(x_1, \alpha_1, \beta_1), \ldots, s_{inv}(x_p, \alpha_p, \beta_p))', \tag{S3-7}$$

$$s_{inv}(x, \alpha, \beta) = \frac{\sqrt[3]{2}p}{\sqrt[3]{-q + \delta}} + \frac{\sqrt[3]{-q + \delta}}{\sqrt[3]{2}} - \frac{l_1}{3k_2}, \tag{S3-8}$$

$$\delta = \sqrt{q^2 + 4p^3}, \tag{S3-9}$$

$$p = \frac{3k_1k_2 - l_1^2}{9k_2^2}, \tag{S3-10}$$

$$q = \frac{2l_1^3 - 9k_1k_2l_1 + 27k_2^2(-l_1 - x)}{27k_2^3}. \tag{S3-11}$$

References

[1] Zhao, Y., Lu, Z.: Fourth-moment standardization for structural reliability assessment. Journal of Structural Engineering **133**(7), 916–924 (2007)
