# Supplementary material for: Genomic data assimilation using a higher moment filtering technique for restoration of gene regulatory networks
Source: BMC Syst Biol. 2015 Mar 13;9:14. doi: 10.1186/s12918-015-0154-2 (PMC4371723; doi:10.1186/s12918-015-0154-2)
Supplement: Additional file 2 — The procedure of the smoothing step in HMEnPF. The procedure of the smoothing step in HMEnPF is described in the file. [file 12918_2015_154_MOESM2_ESM.pdf]

Additional File 2

The smoothing step in HM-EnPF

Here, we assume $\mathcal{T}_{obs} = \mathcal{T}$ for simplicity. The smoothing step also consists of the following three sub-steps. At tth ($t \in \mathcal{T}_{obs}$) time step, for $s = t+1, \ldots, T$,

1. Particle Filter Step
   (a) Resample $\hat{\boldsymbol{x}}_{t|s}^{(n)}$ according to

$$p(\boldsymbol{x}_t|\boldsymbol{Y}_s) = \frac{1}{\sum_{\acute{n}} p(\boldsymbol{y}_s|\boldsymbol{x}_{s|s-1}^{(\acute{n})})} \sum_{n=1}^{N} p(\boldsymbol{y}_s|\boldsymbol{x}_{s|s-1}^{(n)})\delta(\boldsymbol{x}_t - \boldsymbol{x}_{t|s-1}^{(n)}), \tag{S1-1}$$

   where $\delta(\cdot)$ is a Dirac delta function.

   (b) Calculate first and second moments $\mu_{t|s} = E[\{\hat{\boldsymbol{x}}_{t|s}^{(n)}\}]$ and $V_{t|s} = Var[\{\hat{\boldsymbol{x}}_{t|s}^{(n)}\}]$, respectively.

   (c) Standardize $\hat{\boldsymbol{x}}_{t|s}^{(n)}$ as

$$\hat{\boldsymbol{z}}_{t|s}^{(n)} = V_{t|s}^{-\frac{1}{2}} \cdot (\hat{\boldsymbol{x}}_{t|s}^{(n)} - \boldsymbol{\mu}_{t|s}). \tag{S1-2}$$

   (d) Calculate third and fourth central moments $\hat{\boldsymbol{m}}_{t|s}^{(3)} = E[\{\hat{\boldsymbol{z}}_{t|s}^{(n)}\}^3]$ and $\hat{\boldsymbol{m}}_{t|s}^{(4)} = E[\{\hat{\boldsymbol{z}}_{t|s}^{(n)}\}^4]$, respectively.

2. Ensemble Kalman Filter Step
   (a) Calculate Kalman gain

$$K_s = \frac{1}{N-1}\{\sum_{n=1}^{N}(\boldsymbol{x}_{t|s-1}^{(n)} - E[\{\boldsymbol{x}_{t|s-1}^{(n)}\}])(\boldsymbol{x}_{s|s-1}^{(n)} - E[\{\boldsymbol{x}_{s|s-1}^{(n)}\}])'\}(V_{s|s-1} + R_s)^{-1}. \tag{S1-3}$$

   (b) Calculate $\tilde{\boldsymbol{x}}_{t|s}^{(n)}$ as

$$\tilde{\boldsymbol{x}}_{t|s}^{(n)} = \boldsymbol{x}_{t|s-1}^{(n)} + K_s(\boldsymbol{y}_s - \boldsymbol{x}_{s|s-1}^{(n)} + \boldsymbol{w}_s^{(n)}). \tag{S1-4}$$

   (c) Calculate first and second moments $\tilde{\mu}_{t|s} = E[\{\tilde{\boldsymbol{x}}_{t|s}^{(n)}\}]$ and $\tilde{V}_{t|s} = Var[\{\tilde{\boldsymbol{x}}_{t|s}^{(n)}\}]$, respectively.

   (d) Standardize $\tilde{\boldsymbol{x}}_{t|s}^{(n)}$ as

$$\tilde{\boldsymbol{z}}_{t|s}^{(n)} = \tilde{V}_{t|s}^{-\frac{1}{2}} \cdot (\tilde{\boldsymbol{x}}_{t|s}^{(n)} - \tilde{\boldsymbol{\mu}}_{t|s}). \tag{S1-5}$$

   (e) Calculate third and fourth central moments $\tilde{\boldsymbol{m}}_{t|s}^{(3)} = E[\{\tilde{\boldsymbol{z}}_{t|s}^{(n)}\}^3]$ and $\tilde{\boldsymbol{m}}_{t|s}^{(4)} = E[\{\tilde{\boldsymbol{z}}_{t|s}^{(n)}\}^4]$, respectively.

3. Merging Step
   Calculate $\boldsymbol{x}_{t|s}^{(n)}$ as

$$\boldsymbol{x}_{t|s}^{(n)} = \hat{V}_{t|s}^{\frac{1}{2}} S(\boldsymbol{z}_t^{(n)}, \hat{\boldsymbol{m}}_{t|s}^{(3)}, \hat{\boldsymbol{m}}_{t|s}^{(4)}) + \hat{\boldsymbol{\mu}}_{t|s}, \tag{S1-6}$$

$$\boldsymbol{z}_t^{(n)} = S(\tilde{\boldsymbol{z}}_{t|s}^{(n)}, \tilde{\boldsymbol{m}}_{t|s}^{(3)}, \tilde{\boldsymbol{m}}_{t|s}^{(4)})^{-1}. \tag{S1-7}$$
