# Supplementary material for: Genomic data assimilation using a higher moment filtering technique for restoration of gene regulatory networks
Source: BMC Syst Biol. 2015 Mar 13;9:14. doi: 10.1186/s12918-015-0154-2 (PMC4371723; doi:10.1186/s12918-015-0154-2)
Supplement: Additional file 3 — The detailed solution of the EM-algorithm for the estimation of the parameter values. The detailed solution of the E- and M-steps in the EM-algorithm for HMEnPF are described in the file. [file 12918_2015_154_MOESM3_ESM.pdf]

Additional File 3

EM-algorithm for a state space representation of the combinatorial effect model

In the Expectation-step, $q(\boldsymbol{\theta}|\boldsymbol{\theta}_l)$ is calculated by

$$\begin{aligned} q(\boldsymbol{\theta}|\boldsymbol{\theta}_l) = &-\frac{1}{2}\mathrm{tr}\{V_{0|0}^{-1}(V_{0|t} + (\boldsymbol{x}_{0|t} - \boldsymbol{\mu}_0)(\boldsymbol{x}_{0|t} - \boldsymbol{\mu}_0)')\} - \frac{1}{2}\log|\Sigma_{0|t}| \\ &- \frac{1}{2}\sum_{t=1}^{T}\mathrm{tr}\{Q^{-1}E[(\boldsymbol{x}_t - F\boldsymbol{x}_{t-1} - B\mathrm{vec}(\boldsymbol{x}_{t-1}\boldsymbol{x}_{t-1}') - G\boldsymbol{d}_{t-1} - \boldsymbol{u}) \\ &\cdot(\boldsymbol{x}_t - F\boldsymbol{x}_{t-1} - B\mathrm{vec}(\boldsymbol{x}_{t-1}\boldsymbol{x}_{t-1}') - G\boldsymbol{d}_{t-1} - \boldsymbol{u})'|Y_T]\} \\ &- \frac{T}{2}\log|Q| - \frac{1}{2}\mathrm{tr}\{R^{-1}\sum_{t=1}^{T}\{(\boldsymbol{y}_t - \boldsymbol{x}_{t|t})(\boldsymbol{y}_t - \boldsymbol{x}_{t|t})' + V_{t|t}')\} - \frac{T}{2}\log|R| - N(T + \frac{1}{2})\log 2\pi. \end{aligned} \quad \text{(S2-1)}$$

In the Maximization-step, $\boldsymbol{\theta}_l$ is updated to $\boldsymbol{\theta}_{l+1} = \arg\max_{\boldsymbol{\theta}} q(\boldsymbol{\theta}|\boldsymbol{\theta}_l)$. At first, set conditional expectations of $\boldsymbol{x}_t$ as

$$V_t = \frac{1}{N}\sum_{t\in\mathcal{T}}\sum_{n=1}^{N}\boldsymbol{x}_{t|T}^{(n)}\boldsymbol{x}_{t|T}^{(n)'}, \quad \text{(S2-2)}$$

$$V_{lag} = \frac{1}{N}\sum_{t\in\mathcal{T}}\sum_{n=1}^{N}\boldsymbol{x}_{t|T}^{(n)}\boldsymbol{x}_{t-1|T}^{(n)'}, \quad \text{(S2-3)}$$

$$V_{t-1} = \frac{1}{N}\sum_{t\in\mathcal{T}}\sum_{n=1}^{N}\boldsymbol{x}_{t-1|T}^{(n)}\boldsymbol{x}_{t-1|T}^{(n)'}, \quad \text{(S2-4)}$$

$$\Phi_{lag} = \frac{1}{N}\sum_{t\in\mathcal{T}}\sum_{n=1}^{N}\boldsymbol{x}_{t|T}^{(n)}\mathrm{vec}(\boldsymbol{x}_{t-1|T}^{(n)}\boldsymbol{x}_{t-1|T}^{(n)'})', \quad \text{(S2-5)}$$

$$\Phi_{t-1} = \frac{1}{N}\sum_{t\in\mathcal{T}}\sum_{n=1}^{N}\boldsymbol{x}_{t-1|T}^{(n)}\mathrm{vec}(\boldsymbol{x}_{t-1|T}^{(n)}\boldsymbol{x}_{t-1|T}^{(n)'})', \quad \text{(S2-6)}$$

$$\Psi_{t-1} = \frac{1}{N}\sum_{t\in\mathcal{T}}\sum_{n=1}^{N}\mathrm{vec}(\boldsymbol{x}_{t-1|T}^{(n)}\boldsymbol{x}_{t-1|T}^{(n)'})\mathrm{vec}(\boldsymbol{x}_{t-1|T}^{(n)}\boldsymbol{x}_{t-1|T}^{(n)'})', \quad \text{(S2-7)}$$

$$E_{lag} = \frac{1}{N}\sum_{t\in\mathcal{T}}\sum_{n=1}^{N}\boldsymbol{x}_{t|T}^{(n)}\boldsymbol{d}_{t-1}', \quad \text{(S2-8)}$$

$$E_{t-1} = \frac{1}{N}\sum_{t\in\mathcal{T}}\sum_{n=1}^{N}\boldsymbol{x}_{t-1|T}^{(n)}\boldsymbol{d}_{t-1}', \quad \text{(S2-9)}$$

$$E_{t-1}^2 = \frac{1}{N}\sum_{t\in\mathcal{T}}\sum_{n=1}^{N}\mathrm{vec}(\boldsymbol{x}_{t-1|T}^{(n)}\boldsymbol{x}_{t-1|T}^{(n)'})\boldsymbol{d}_{t-1}', \quad \text{(S2-10)}$$

$$\boldsymbol{z} = \sum_{t\in\mathcal{T}}^{T} \boldsymbol{d}_{t-1}, \tag{S2-11}$$

$$Z = \sum_{t\in\mathcal{T}}^{T} \boldsymbol{d}_{t-1}\boldsymbol{d}'_{t-1}. \tag{S2-12}$$

$$\boldsymbol{s}_t = \frac{1}{N}\sum_{t\in\mathcal{T}}\sum_{n=1}^{N} \boldsymbol{x}_{t|T}^{(n)}, \tag{S2-13}$$

$$\boldsymbol{s}_{t-1} = \frac{1}{N}\sum_{t\in\mathcal{T}}\sum_{n=1}^{N} \boldsymbol{x}_{t-1|T}^{(n)}, \tag{S2-14}$$

$$\boldsymbol{s}_{t-1}^2 = \frac{1}{N}\sum_{t\in\mathcal{T}}\sum_{n=1}^{N} \mathrm{vec}(\boldsymbol{x}_{t-1|T}^{(n)}\boldsymbol{x}_{t-1|T}^{(n)'}). \tag{S2-15}$$

Let $\boldsymbol{v}_{lag,i}$, $\boldsymbol{\phi}_{lag,i}$ and $\boldsymbol{\phi}_{t-1,i}$ be a transpose of the ith row vector of V_{lag}, Φ_{lag} and Φ_{t-1}, respectively. Then, $\boldsymbol{\theta}$ is updated as

$$\boldsymbol{a}_i^{\mathcal{A}_i} = V_{t-1}^{\mathcal{A}_i^{-1}}(\boldsymbol{v}_{lag,i}^{\mathcal{A}_i} - \phi_{t-1}^{\mathcal{A}_i\times\mathcal{B}_i}\boldsymbol{b}_i^{\mathcal{B}_i} - E_{t-1}^{\mathcal{A}_i\times\mathcal{G}_i}\boldsymbol{g}_n^{\mathcal{G}_i} - u_i\boldsymbol{s}_{t-1}^{\mathcal{A}_i}), \tag{S2-16}$$

$$\boldsymbol{b}_i^{\mathcal{B}_i} = \Psi_{t-1}^{\mathcal{B}_i^{-1}}(\boldsymbol{\phi}_{lag,i}^{\mathcal{B}_i} - \phi_{t-1}^{\mathcal{A}_i\times\mathcal{B}_i'}\boldsymbol{a}_i^{\mathcal{A}_i} - E_{t-1}^{2^{\mathcal{B}_i}\times\mathcal{G}_i}\boldsymbol{g}_i^{\mathcal{G}_i} - u_i\boldsymbol{s}^{2^{\mathcal{B}_i}}), \tag{S2-17}$$

$$\boldsymbol{g}_i^{\mathcal{G}_i} = Z^{\mathcal{G}_i^{-1}}(\boldsymbol{e}_{lag,n}^{\mathcal{G}_i} - E_{t-1}^{\mathcal{A}_i\times\mathcal{G}_i'}\boldsymbol{a}_n^{\mathcal{A}_i} - E_{t-1}^{2^{\mathcal{B}_i}\times\mathcal{G}_i'}\boldsymbol{b}_i^{\mathcal{B}_i} - u_n\boldsymbol{z}^{\mathcal{G}_i}) \tag{S2-18}$$

$$\boldsymbol{u} = \frac{\boldsymbol{s}_t - A\boldsymbol{s}_{t-1} - B\boldsymbol{s}^2 - G\boldsymbol{z}}{T}, \tag{S2-19}$$

$$Q = \frac{1}{T}\sum_{t=1}^{T} E[(\boldsymbol{x}_t - A\boldsymbol{x}_{t-1} - B\mathrm{vec}(\boldsymbol{x}_{t-1}\boldsymbol{x}'_{t-1}) - G\boldsymbol{d}_{t-1} - \boldsymbol{u}) \cdot (\boldsymbol{x}_t - A\boldsymbol{x}_{t-1} - B\mathrm{vec}(\boldsymbol{x}_{t-1}\boldsymbol{x}'_{t-1}) - G\boldsymbol{d}_{t-1} - \boldsymbol{u})'|Y_T], \tag{S2-20}$$

$$\boldsymbol{\mu}_0 = \boldsymbol{x}_{0|T}, \tag{S2-21}$$

$$R = \frac{1}{T}\sum_{t\in\mathcal{T}_{obs}} \{(\boldsymbol{y}_t - \boldsymbol{x}_{t|t})(\boldsymbol{y}_t - \boldsymbol{x}_{t|t})' + V_{t|t}\}, \tag{S2-22}$$

where $\mathcal{A}$, $\mathcal{B}$ and $\mathcal{G}$ are active sets of elements for A, B and G, respectively. For example, $\boldsymbol{a}_i^{\mathcal{A}}$ is an $|\mathcal{A}|$-dimensional vector consisting of elements regulating the ith gene.
