# Supplementary material for: Genomic data assimilation using a higher moment filtering technique for restoration of gene regulatory networks
Source: BMC Syst Biol. 2015 Mar 13;9:14. doi: 10.1186/s12918-015-0154-2 (PMC4371723; doi:10.1186/s12918-015-0154-2)
Supplement: Additional file 4 — The functions measuring the effectiveness of regulatory edges. The functions measuring the effectiveness of regulatory edges e a, e b and e g are described in the file. [file 12918_2015_154_MOESM4_ESM.pdf]

Additional File 4

The function for measuring the effectiveness of regulation

Set the functions for measuring the effectiveness of individual, combinatorial and drug effects on to the ith gene under the model $\mathcal{M}$ as

$$e_a(\mathcal{M}, i) = \boldsymbol{a}_i' V_{t-1} \boldsymbol{a}_i - 2\boldsymbol{v}_{lag,i} \boldsymbol{a}_i + 2\boldsymbol{b}_i' \phi_{t-1}' \boldsymbol{a}_i + 2\boldsymbol{g}_i' E_{t-1}' \boldsymbol{a}_i + 2u_i \boldsymbol{s}_{t-1}' \boldsymbol{a}_i, \tag{S3-1}$$

$$e_b(\mathcal{M}, i) = \boldsymbol{b}_i' \Psi_{t-1} \boldsymbol{b}_i - 2\boldsymbol{\phi}_{lag,i}' \boldsymbol{b}_i + 2\boldsymbol{a}_i' \phi_{t-1} \boldsymbol{b}_i + 2\boldsymbol{g}_i' E_{t-1}^{2'} \boldsymbol{b}_i + 2u_i \boldsymbol{s}_{t-1}^{2'} \boldsymbol{b}_i, \tag{S3-2}$$

$$e_g(\mathcal{M}, i) = \boldsymbol{g}_i' Z \boldsymbol{g}_i - 2\boldsymbol{e}_{lag,i}' \boldsymbol{g}_i + 2\boldsymbol{a}_i' E_{t-1} \boldsymbol{g}_i + 2\boldsymbol{b}_i' E_{t-1}^{2} \boldsymbol{g}_i + 2u_i \boldsymbol{z}' \boldsymbol{g}_i, \tag{S3-3}$$

respectively. It should be noted that the functions are derived when calculating $\arg\max_{\boldsymbol{a}_i} q(\boldsymbol{\theta}|\boldsymbol{\theta}_l)$, $\arg\max_{\boldsymbol{b}_i} q(\boldsymbol{\theta}|\boldsymbol{\theta}_l)$ and $\arg\max_{\boldsymbol{g}_i} q(\boldsymbol{\theta}|\boldsymbol{\theta}_l)$, respectively.
