# Supplementary material for: Genomic data assimilation using a higher moment filtering technique for restoration of gene regulatory networks
Source: BMC Syst Biol. 2015 Mar 13;9:14. doi: 10.1186/s12918-015-0154-2 (PMC4371723; doi:10.1186/s12918-015-0154-2)
Supplement: Additional file 6 — The corticosteroid pharmacokinetics/dynamics in rat skeletal muscle. The differential equations of rat pharmacokinetics/dynamics are described in the file. [file 12918_2015_154_MOESM6_ESM.pdf]

Additional File 6

Corticosteroid Pharmacokinetics/dynamics

Because corticosteroid pharmacokinetics/dynamics in skeletal muscle have been modeled based on differential equations by Yao *et al.* (2008) as shown in Fig. **??**, the time-dependent concentration of corticosteroid in nucleus in rat skeletal muscle can be obtained as followings;

$$\frac{d\mathrm{mRNA_R}(t)}{dt} = k_{s\_Rm} \cdot \left\{1 - \frac{\mathrm{DR_N}(t)}{\mathrm{IC_{50\_Rm}} + \mathrm{DR_N}(t)}\right\} - k_{d\_Rm} \cdot \mathrm{mRNA_R}(t), \tag{S3-1}$$

$$\frac{d\mathrm{R}(t)}{dt} = k_{s\_R} \cdot \mathrm{mRNA_R}(t) + R_f \cdot k_{re} \cdot \mathrm{DR_N}(t) - k_{on} \cdot \mathrm{D}(t) \cdot \mathrm{R}(t) - k_{d\_R} \cdot \mathrm{R}(t), \tag{S3-2}$$

$$\frac{d\mathrm{DR}(t)}{dt} = k_{on} \cdot \mathrm{D}(t) \cdot \mathrm{R}(t) - k_T \cdot \mathrm{DR}(t), \tag{S3-3}$$

$$\frac{d\mathrm{DR_N}(t)}{dt} = k_T \cdot \mathrm{DR}(t) - k_{re} \cdot \mathrm{DR_N}(t), \tag{S3-4}$$

where $\mathrm{mRNA_R}(t)$ is the concentration of mRNA of the receptor protein, $\mathrm{R}(t)$ is the concentration of the receptor protein, $\mathrm{DR}(t)$ is the concentration of the drug-receptor complex, $\mathrm{DR_N}(t)$ is the concentration of the drug-receptor complex in nucleus, and *Synthesis* and *Degradation* mean synthesis and degradation processes, respectively. $\mathrm{DR_N}(t)$ was used for $\boldsymbol{d}_t$. These parameter values, $k_{s\_Rm}, \mathrm{IC_{50\_Rm}}, k_{d\_Rm}$, k_{s_R}, k_{d_R}, R_f, $k_{re}, k_{on}, k_{d\_R}, k_T$, are shown in Table 1. According to Sun *et al.* (1998), the time-evolution of the plasma concentration of corticosteroid is given as

$$\mathrm{D}(t) = 39,130 \cdot e^{-7.54t} + 12,670 \cdot e^{-1.20t}. \tag{S3-5}$$

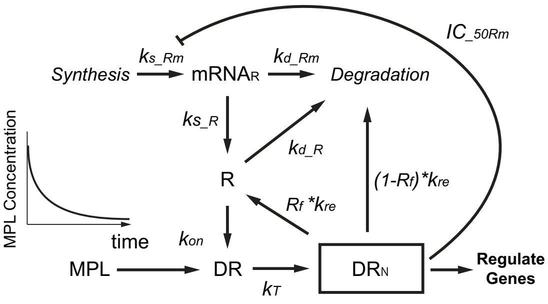

Figure 1: Corticosteroid Pharmacokinetics/dynamics in rat skeletal muscle. The pharmacokinetics/dynamics developed by Yao *et al.* (2008). The dynamics behavior of the concentration of biomolecules is described by differential equations. $\mathrm{mRNA_R}$ is the concentration of mRNA of the receptor protein, R is the concentration of the receptor protein, DR is the concentration of the drug-receptor complex, $\mathrm{DR_N}$ is the concentration of the drug-receptor complex in nucleus, and *Synthesis* and *Degradation* mean synthesis and degradation processes, respectively.

Table 1: **The values of the parameters for corticosteroid pharmacodynamics illustrated in Fig. ??.**

parameter	value
k_{s_Rm}(fmol/g/h)	0.416
k_{d_Rm}(1/h)	0.139
k_{s_R}(fmol/g/h)	0.777
k_{d_R}(1/h)	0.0356
k_{on}(1/nmol/h)	0.00269
k_T(1/T)	90
k_{re}(1/h)	0.618
R_f	0.720
$IC_{\_50Rm}$(fmol/mg)	0.911
GR^0_{mRNA}(fmol/g)	2.99
GR^0(fmol/mg)	65.3
